# The refugee and migrant health “global competency standards for health workers”: results of a survey in general practitioner trainees in Sicily

**DOI:** 10.3389/fpubh.2024.1392025

**Published:** 2024-10-15

**Authors:** Livia Cimino, Alessandra Pirrello, Alessandra Casuccio, Claudio Costantino, Davide Graci, Nicolò Piazza, Palmira Immordino

**Affiliations:** ^1^U.O.C. Igiene degli Ambienti di Vita, Azienda Sanitaria Locale (ASP) Palermo, Palermo, Italy; ^2^Department of Health Promotion, Mother and Childcare, Internal Medicine and Medical Specialties "G. D'Alessandro", University of Palermo, Palermo, Italy

**Keywords:** competency frameworks, public health, cultural sensitivity, health service utilisation, migrants, refugees, cultural competence

## Abstract

**Background:**

Refugees and migrants may represent the most vulnerable communities in many societies. Health systems should be sensitive to needs of refugees and migrants. The document “The Refugee and Migrant Health: Global Competency Standards for Health Workers (the *Standards*)” identifies the competencies and areas of practice of health workers working with refugees and migrants. The aim of this study is to provide an analysis of these competences and training needs, identifying the educational priorities for the implementation of the *Standards* in Sicily, Italy.

**Methods:**

A cross-sectional analytical pilot study was conducted by administering a questionnaire, in electronic format “Google Form ®,” to doctors attending the Medical Training Course for General Practitioners in Sicily (Italy). Data obtained were collected in a Microsoft Excel database and analyzed with IBM SPSS Software 24 version. Absolute and relative frequencies were calculated for relevant categorical variables. Univariate analysis of the selected variables was subsequently carried out. The significance level chosen was a *p* value <0.05 (two-tailed).

**Results:**

A total of 192 General Practitioner (GP) trainees responded to the questionnaire. They were asked if their training course comprised a Global Health Course that included the topic of health protection and social and health care for migrant populations and the 65.4% of them answered “No” or “Do not Know.” GP trainees were also asked if they considered useful to include a Global Health Course dedicated to the management of patients with a migratory background within the training course in general medicine. Of the total 192 participants, 81.9% answered “Yes.” Overall, in a simple regression model, the perception of having addressed migrants’ health needs is positively correlated with having attended a Global Health Course (OR = 3.34 95%CI 1.2–9.1; *p* = 0.018).

**Conclusion:**

This study identified educational priorities for the implementation of the *Standards* in Sicily for doctors attending the Medical Training Course for General Practitioners. We hope that the results of this study will guide and inform possible future projects to implement the *Standards* at a national level.

## 1 Introduction

Refugees and migrants may represent the most vulnerable community in many societies. Too often they live in conditions of low security, on the fringes of society, in fear of deportation and without access to a reasonable level of essential services, including health services. They may suffer discrimination, social exclusion, negative attitudes, and stigmatizing stereotypes. All countries should aspire to build strong health systems, supported by a well-trained, culturally sensitive, and competent health workforce capable of responding to the needs of all. In this paper we refer, unless otherwise specified, to both groups of migrants and refugees. Regarding the definition of the two categories, the definition of a refugee is outlined in Article 1 of the 1951 Convention Relating to the Status of Refugees, which states: “For the purposes of this Convention, the term ‘refugee’ shall apply to any person … because of well-founded fear of being persecuted for reasons of race, religion, nationality, membership of a particular social group or for his political opinions, is outside the country of his citizenship and cannot or, due to this fear, does not want to avail himself of the protection of that country; or who, not having a nationality and being outside the country of his previous habitual residence as a result of such events, is unable or, due to such fear, is unwilling to return to it (1).” The term “migrant,” according to the International Organization for Migration (IOM), is a generic term not defined by international law, and describes a person who moves away from their usual place of residence either permanently or temporarily for reasons related to work, economic, educational, or other motives, whether or not they are present in the host country (2). Therefore, the differences concern the reasons for moving, legal status, and rights to assistance and protection.

Migrants are granted a different legal status based on the country in which they stay, which may have different interpretations regarding the right and access to essential health services within a given national legislation. However, under international law, such access remains universal for all, in line with the 2030 Agenda for Sustainable Development (SDGs), with Sustainable Development Goal 3 (ensure healthy lives and promote well-being for all at all ages) (3). Health systems should be sensitive to the specific needs of refugees and migrants, also considering the peculiarities of their health problems. Comprehensive national health policies, supported by legislative frameworks and financial efforts, are therefore needed. Furthermore, the need to define and identify consistent standards of competence in practice for health workers providing services to refugees and migrants is becoming increasingly evident. The WHO Health and Migration Department, in collaboration with the “WHO Health Workforce Department,” in 2021 identified the need to specifically personalize competences to respond to the different health needs of refugee and migrant populations, issuing the document “The Refugee and Migrant Health: Global Competency Standards for Health Workers (the *Standards*)” (4). This document is strongly aligned with the Global Competency Framework for Universal Health Coverage, developed by the WHO Health Workforce Department to identify the competencies and areas of practice of health workers in primary healthcare, but contains additional specifications for minimum behavioral standards and evidence of effectiveness (5). Competence can be defined as the ability to perform work tasks to a defined standard. It is multidimensional and dynamic, changing with time, experience, and environment, and is equivalent to possessing the competences necessary to carry out specific activities in each context (5, 6). The health workforce competencies and behaviors identified in the *Standards* are organized into five key domains. These are presented in a way that they can be applied to the context and environment in which healthcare workers carry out their work, taking into consideration the requirements and constraints of the local healthcare system and, equally, the peculiar characteristics of the migrant and refugees populations. Countries with a sizable presence of refugees and migrants have adapted their healthcare systems in various ways to meet the needs of these populations. General practitioners (GPs) are the first to come into contact with migrants and are also the first to interpret their needs and direct them towards a correct use of the National Health System (NHS). They play a pivotal role in addressing the healthcare needs of these population groups in Sicily, a region that has seen a significant influx of people due to its geographical proximity to North Africa and the Middle East. These primary care providers serve as the first point of contact within the healthcare system, offering essential services such as initial health assessments, treatment of common illnesses, and management of chronic conditions. GPs are crucial in identifying and addressing specific health issues prevalent among migrants, including infectious diseases, mental health concerns, and the effects of trauma. They also play a vital role in facilitating access to further medical services and coordinating care with specialists. By ensuring continuity of care and integrating health services, GPs may contribute significantly to the overall well-being and integration of migrants into the local.

The aim of this study is to provide an analysis of the competences and training needs regarding the health of migrants and refugees, identifying the educational priorities for the implementation of the *Standards* in Sicily. This study is structured as a pilot study at regional level with the intention of conducting a competence mapping at national level and then proceeded, based on the results obtained, with the implementation of the *Standards*. Doctors attending the Medical Training Course for General Practitioners (herein after referred to as “General Practitioner (GP) Trainees”) in the Sicily region (Italy) were identified as the first target for piloting this project. These doctors were asked to respond to a questionnaire regarding healthcare competencies in the care of migrants. The aim was to highlight the critical issues that limit access to care and, above all, to identify potential actions in the field of training as well as political interventions that could address this inequality. Ultimately aims at strengthening the capacity of healthcare personnel through a competences-based approach to provide quality people-centered and culturally sensitive health services to refugees and migrants. The results of this study will guide and inform possible future projects at national level.

## 2 Materials and methods

In order to map existing competencies, evaluate the educational programs provided and assess the perception of health workers regarding the need for specific courses dedicated to the health of migrants and refugees, a cross-sectional analytical pilot study was conducted through the administration of a questionnaire in electronic format “Google Form ®.” The questionnaire was administered to GP Trainees, identified as the first target group for this study. GP Trainees were recruited with the support of the Teaching Directorate of the General Medicine Course for the Sicily Region which took care of sending the dedicated link, created on Google Form ® platform, to the mailing list of those enrolled. The questionnaire was administered in Italian. The purpose of the study, the treatment methods and the storage and protection of personal data were explained, and the informed consent form was signed and collected. The total number of subjects involved in the study is 275 in the triennium 2021/2024 and includes GP trainees of both sexes who, on a voluntary basis, decided to complete the administered questionnaire. Data was collected from 06/15/2023 to 08/25/2023. A minimum sample size of 161 subjects was calculated to provide 95% power with α = 0.05.

The study was approved on 19 April 2023 by the Palermo 1 Ethics Committee. The decision to involve general practitioners in training involves several factors, primarily the fact that their education includes primary care, preventive medicine, and the management of chronic diseases. This leads us to believe that future general practitioners need to handle a wide range of health issues, including those affecting migrant and refugee populations. Furthermore, general practitioners often serve as the first point of contact with the national healthcare system. By focusing on GPs, we can gain insights into how primary care practices can be enhanced to better serve migrant and refugee populations.

### 2.1 The questionnaire

Participants filled out a questionnaire consisting of 38 questions divided into 5 sections. In the first section (5 questions) socio-demographic aspects are investigated (age, sex, year of the course) and any professional and training experience regarding health and migration issues (frequency of assistance to patients with a migratory background). The second section (9 questions) explored information relating to Global Health courses focusing on the protection of the health of migrants and refugees already attended by the participants and in particular: title of the Course, surname and name of the person in charge of the Course, role of the person in charge of the Course, number of teaching hours, number of credits, year of training in which the Course is included, courses or activities relating to the aforementioned topics outside the university context (Name of the course and body or association responsible for the course). The third section (5 questions) is based on the “Competency Standards” and collects the perception and views healthcare professionals have on their intercultural competencies. Furthermore, the healthcare worker is asked how much he/she deems appropriate and useful for the purpose of his/her profession to acquire specific competences on the management of patients with a migratory background. The fourth section (14 questions) includes multiple choice questions in which the healthcare professional is asked how frequently he believes he meets the health needs of patients with a migratory background. There are also multiple-choice questions by which participants are asked to indicate to what extent they agree or disagree with a series of general statements regarding the way they try to meet their health needs or how much often their competences are in line with good culturally sensitive care practices. In the last section (5 questions) participants are asked to express an opinion regarding the difficulties that can be encountered when the healthcare worker and patient have a different cultural background, and the usefulness of including a course dedicated to the management of patients with migratory background.

### 2.2 Statistical analysis

The data obtained were collected in a Microsoft Excel database, automatically compiled by the Google Form® online system. Absolute and relative frequencies have been reported for qualitative variables and means and standard deviation (SD) for quantitative variables. In order to assess which factors were associated with the perceived health needs of migrant populations, we used the Pearson’s chi-square test and Fisher exact test, as needed.

Furthermore, a bivariable regression model was used to examine the correlations between perception of having addressed migrants’ health needs or considering usefulness of including a Global Health Course (dependent variables), and some characteristics of GP trainees such as age, sex, having completed specific training course or clinical practice with people with different cultural background (independent variables). Odds ratios and related 95% confidence intervals (95% CI) were reported as well as the *p*-value. A multivariable regression model was used to analyze the factors associated with usefulness of including a Global Health Course in the training and evaluate the covariates associated at bivariable analysis with a *p* value equal or lower than 0.05. Adjusted OR and related 95% confidence intervals (95% CI) were reported as well as the *p*-value.

Data were analyzed by IBM SPSS Software 24 version (IBM Corp., Armonk, NY, USA). All *p*-values were two-sided and *p* ≤ 0.05 was considered statistically significant.

## 3 Results

A total of 192 GP Trainees (70% of overall population) filled the questionnaire, administered in the period between 06/15/2023 and 08/25/2023, of which 144 were attending the second and 48 the third year of the Medical Training Course for General Practitioners (75 and 25% respectively). Table 1 reports the characteristics of the study population. The average age of the population is 36.1 (±6.1) years and includes 77 (40.2%) males and 115 (59.8%) females. When asked about the frequency with which they had to deal with patients with a migratory background in clinical practice, 32 (16.6%) GP trainees answered “Never,” 46 (23.9%) answered “Almost never,” 65 (33.8%) responded “Sometimes” (at least once every 3 months), 36 (18.7%) responded “Often” and 13 (6.7%) responded “Always” (more than once a month). When the residents were asked if their training course included a Global Health Course that covered health protection and social and health care for migrant populations, 65 (33.8%) responded “Yes,” 40 (20.8%) “No” and the largest percentage (45.3%) responded that they did not know. The average number of teaching hours dedicated to global health was 6.3(±3.1) for a total of 7.6 (1.2) credits (Table 2).

**Table 1:** Characteristics of the study population.

Mean age ± SD	36.1 ± 6.1
Sex	
Male	77 (40.1%)
Female	115 (59.8%)
Year of course	
Second	144 (75.3%)
Third	48 (25.3%)
Have you ever, in clinical practice, had to deal with individuals with a migratory background?	
Never	32 (16.6%)
Almost never (1–2 times a year)	46 (23.9%)
Sometimes (at least one every 3 months)	65 (33.8%)
Often (at least once a month)	36 (18.7%)
Always (more than once a month)	13 (6.7%)
Is there a Global Health Course in your Training Course that includes the topic of health protection and socio-health care for migrant populations?	
Yes	65 (33.8%)
No	40 (20.8%)
Do not know	87 (45.3%)

**Table 2:** Global Health Courses.

Course information	
Average number of teaching hours	6.3(±3.1)
Average number of credits	7.6(1.2)

In the third section (Table 3) of the questionnaire, GP trainees were asked to express their level of agreement with some statements based on the document “Competency Standards,” on a scale from 1 to 5. Most of the interviewees said they “strongly agreed” with the statements that concerned the importance of considering the cultural background of the patient in treating her/him, the cultural sensitivity and the ability to communicate in an intercultural perspective as a value for providing better healthcare services, the usefulness, for the purpose of the profession, of acquiring specific competencies on the management of patients with a migratory background and the possibility for the Specific Training within the Medical Training Course for General Practitioners to provide deeper knowledge regarding the aforementioned topic.

**Table 3:** Competency standards.

Indicate how much you agree with the following sentences: by giving a grade from 1 to 5. (1 means “I do not agree at all” and 5 means “I strongly agree”).						
	1	2	3	4	5	Do not Know
Health service providers should consider a patient’s cultural background when treating him/her.	4 (2.0%)	0	23 (11.9%)	46 (23.9%)	113 (58.8%)	6 (3.1%)
Cultural awareness is important in providing best-practice health care.	2 (1%)	1 (0.5%)	12 (6.25%)	39 (20.3%)	131 (68.2%)	6 (3.1%)
Being able to effectively communicate cross-culturally with patients is important to best practice health care.	0	1 (0.5%)	11 (5.7%)	36 (18.7%)	138 (71.8%)	6 (3.1%)
It could be useful for my profession to acquire specific skills on the management of people with a migratory background.	1 (0.5%)	1 (0.5%)	21 (10.9%)	42 (21.8%)	121 (63.0%)	6 (3.1%)
The training course in general practice should provide me more professional knowledge to interact more appropriately with people with a migratory background.	3 (1.5%)	4 (2%)	26 (13.5%)	54 (28.1%)	97 (50.5%)	8 (4.1%)

Table 4 instead shows questions related to the resident’s perception of addressing the health needs of people with migratory background. Half of those interviewed reported that only sometimes they felt being able to address the health needs of patients with a different cultural background. More than 50% of those interviewed also responded that they found it more difficult to get in touch with patients with a different cultural background while 20.8% reported that this was often the case. The vast majority of those interviewed (69.2%) support universal access to quality health care, irrespective of the person’s legal status and related legal, administrative, and financial barriers to access. More than the half of the GP Trainees (respectively 49.4 and 68.2%) strongly agrees with assisting refugees and migrants to develop their awareness of the right to health. They also agree on supporting them to improve their knowledge of and ability to navigate, the host country’s health system and that patients have the right to receive timely, gender- and age-appropriate information, including assistance with communication (Table 5).

**Table 4:** Addressing the health needs of those with different cultural background.

Question	Always	Often	Sometimes	Rarely	Never	Do not know
Do you feel to attend health needs of patients from different cultural backgrounds?	12(6.2%)	52(27%)	96(50%)	17(8.8%)	1(0.5%)	14 (7.2%)
Do you find it more difficult to come into contact with patients with a cultural background different from yours?	4(2%)	40(20.8%)	100(52%)	31(16.1%)	9(4.6%)	8(4.1%)
Do you feel that your cultural background makes some patients from a different cultural background uncomfortable?	4(2%)	16(8.3%)	64(23.9%)	51(26.5%)	44(22.9%)	13(6.7%)

How do you feel that you meet the health needs of patients with a different cultural background? Indicate how much you agree with the following sentences by giving a grade from 1 to 5. (1 means “I do not agree at all” and 5 means “I strongly agree”).					
	1	2	3	4	5
I adapt practice to the needs of the person in view of their migration and displacement experiences, taking into consideration the impact of these experiences on access to health care, including barriers to access.	4 (2%)	2 (1%)	43 (22.3%)	71 (36.9%)	72(37.5%)
I Support universal access to quality health care, irrespective of the person’s legal status and related legal, administrative and financial barriers to access.	3 (1.5%)	2 (1%)	13 (6.7%)	41 (21.3%)	133 (69.2%)
I assess the level of education and health literacy of the patient with a migratory background.	1(0.5%)	3(1.5%)	927 (14%)	68 (35.4%)	92 (47.9%)
I assist refugees and migrants to develop their awareness of the right to health by supporting them improve their knowledge of, and ability to navigate, the host country’s health system.	2 (1%)	1 (0.5%)	33 (17.1%)	61 (31.7%)	95 (49.4%)
Patients have the right to receive timely, gender- and age-appropriate information, including assistance with communication.	1 (0.5%)	1 (0.5%)	15 (7.8%)	44 (22.9%)	131 (68.2%)
I involve qualified personnel, including interpreters and cultural mediators, as appropriate and/or use linguistic and communicative tools that are culturally appropriate and sensitive.	8 (4.1%)	9 (4.6%)	42 (21.8%)	50 (26%)	83 (43.2%)
I try to communicate in simple language, avoiding the use of medical jargon, and ensure that the patients understand the information about their health care taking into account language, communication and health literacy barriers.	0	1 (0.5%)	18 (9.3%)	37 (19.2%)	136 (70.8%)
When transferring a patient to the care of other caregivers, I include, through verbal and/or written communication, information about individual, cultural and linguistic needs, as well as the factors related to the migration process.	3 (1.5%)	4 (2%)	32 (16.6%)	54 (28.1%)	99 (51.5%)
I recognise that the health needs of refugees and migrants may differ from those of the general population.	0	2 (1%)	20 (10.4%)	59 (26%)	111 (57.8%)
I use evidence-based guidelines and standards, where they exist, to address the specific health needs of migrants and refugees, including mental health and psychosocial support, psychological first aid, pain management and medication management.	6 (3.1%)	3 (1.5%)	37 (19.2%)	58 (30.2%)	88 (45.8%)
I participate in the generation of evidence, where possible, to inform the development of guidelines and standards to respond to health needs of refugees and migrants and support the translation of evidence into practice when providing care to refugees and migrants.	24 (12.5%)	14 (7.2%)	49 (25.5%)	45 (23.4%)	60 (31.2%)
I believe that people with a migratory background may experience discrimination with potential impact on their health status.	9 (4.6%)	9 (4.6%)	35 (18.2%)	51(26.5%)	88 (45.8%)
I continuously adapt clinical practice to meet the needs of patients from a perspective of cultural sensitivity, aware of the impact of culture, beliefs, values and prejudices.	1 (0.5%)	4 (2.0%)	39 (20.3%)	62 (32.2%)	86 (44.7%)

**Table 5:** Usefulness of including a course dedicated to the management of patients with migratory background.

Would you find it useful to include a Global Health Course dedicated to the management of patients with a migratory background within the Medical Training Course for General Practitioners?	
Yes	157 (81.7%)
No	11 (5.7%)
Do not Know	24 (12.5%)
Which training experience would you find most useful?	
Lectures	56 (29.1%),
Professionalisation activities	55 (28.6%)
Intra-network training	57 (29.6%)
Traineeships	24 (12.5%)
Which learning method would you find most useful?	
Live experience with a tutor	100 (52%)
Discussion of cases	32 (16.6%)
Lessons	23 (11.9%)
Simulation	21 (10.9%)
Peer training	10 (5.2%)
Role playing	5 (2.6%)
Narrative approach	4 (2%)
Self-study	4 (2%)
Distance learning	1 (0.5%)

The GP trainees were asked if they considered it useful to include a Global Health Course dedicated to the management of patients with a migratory background within the Medical Training Course for General Practitioners: of the total 192 participants the 81.7% answered “Yes.” Almost 30% of the respondents (29.6%) considered intra network training the most useful training experience and live experience with a tutor the most useful learning method (52%).

Among those GP Trainees reporting previous experiences in dealing with people with a different background (114), doctors’ perception of addressing their health needs is significantly associated with having completed a specific training course on migrant and refugee health (*p* < 0.05).

Overall, in a simple regression model, the perception of having addressed migrants’ health needs is positively correlated with having attended a Global Health Course (OR = 3.34 95%CI 1.2–9.1; *p* = 0.018) and with to clinical practice with people with different cultural background (OR = 3.94 95%CI 1.8–8.9; *p* = 0.001).

These significant correlations were also both confirmed in a multivariable regression model. Regardless of gender and age (Table 6).

**Table 6:** Factors associated with perception of addressing health needs of patients with a different cultural background at bivariable (Crude OR) and multivariable (Adjusted OR) regression analysis (95% CI: 95% confidence interval).

	Perception of addressing health needs of patients with a different cultural background (Yes vs. No)			
	Crude OR (95% CI)	*p*-value	Adj-OR (95% CI)	*p*-value
Global Health Course in the medical training course for general practitioners				
No	Ref.	0.018	Ref.	0.026
Yes	3.34 (1.2–9.1)		3.26 (1.1–9.2)	
Clinical practice with patients with migratory background				
No	Ref.	0.001	Ref.	0.001
Yes	3.94 (1.8–8.9)		3.96 (1.7–9.2)	
Age				
	Ref.	0.448	Ref.	0.574
	1.02 (0.9–1.1)		1.01 (0.9–1.0)	
Sex				
Male	Ref.		Ref.	0.388
Female	0.89 (0.4–1.9)		0.69 (0.2–1.06)	

In a multivariable regression model, among all those interviewed, the inclusion of the course was considered useful by the female GP Trainees (OR 3.0 95%CI 1.4–6.7; *p* = 0.005; Table 7).

**Table 7:** Factors associated with usefulness of including a Global Health Course at multivariable (Adjusted OR) regression analysis. (95% CI: 95% confidence interval).

	Usefulness of including a Global Health Course (Yes vs.No)	
	Adj OR (95% CI)	*p*-value
Global Health Course in the medical training course for general practitioners		
No	Ref.	0.498
Yes	1.35 (0.5–3.2)	
Sex		
Male	Ref.	0.005
Female	3.0 (1.4–6.7)	
Clinical practice with patients with migratory background		
No	Ref.	0.136
Yes	1.8 (0.8–4.0)	
Age		
	Ref.	0.470
	1.02 (0,9–1.0)	
Feeling of attending health needs of patients with a different cultural background		
No	Ref.	0.470
Yes	1.7 (0.6–4.4)	

## 4 Discussion

Europe is currently the leading destination of international migration, with 87 million migrants (30.9% of the international migrant population), followed closely by the 86 million international migrants living in Asia (30.5%). North America is the destination for 59 million international migrants (20.9%), followed by Africa with 25 million migrants (9%) (7).

Italy is a country of entry, transit and in some cases arrival for refugees and migrants; it is easy to understand, given its geographical location, that it represents a key entry point for migrants from Africa who want to reach Europe (8). In (7), a 56% increase in sea arrivals, coupled with unprecedented refugee arrivals from Ukraine and increased land arrivals through Western Balkans, put Italy’s asylum system under pressure. Most sea arrivals departed from Libya, followed by arrivals from Tunisia and Türkiye. Boats carried people mostly originating from Bangladesh, Egypt and Tunisia. 13,487 land arrivals were intercepted at the Italy-Slovenia border in (7), a 44% increase compared to 2021. People mainly originated from Afghanistan, Bangladesh and Pakistan (9, 10). Other migrant populations significantly present in Italy are: Albanians, Moroccans and Romanians (11).

Primary health care (PHC) in Italy is typically provided by GPs, although in recent years there has been a shift towards an integrated and multidisciplinary approach, with GPs and other health professionals moving to group practices (12).

Nevertheless, GPs are essential in meeting the needs of refugees and migrants by providing initial health assessments, managing acute and chronic conditions, and addressing mental health issues. By coordinating with other healthcare providers and social services, GPs should also help integrate migrants into the healthcare system and the broader community, enhancing their overall well-being. Findings from a study conducted in Australia highlighted the critical role GPs play in the resettlement of refugees and the necessity for greater information and resources to assist GPs in managing refugee health effectively. The study also pointed out several social factors that significantly impact the health of refugees, particularly their psychological well-being (13).

Addressing these issues requires comprehensive training for healthcare providers to recognize and mitigate the social determinants of health and to advocate for enhanced mental health services and community support for refugees.

A survey conducted in 2019 evaluated the availability of training opportunities focused on the topic of global health in Italian Schools of Medicine and Surgery (14). A total of 38 Global Health courses have been identified, of which 7 are compulsory and 31 are elective. As regards geographical distribution, 9 courses took place in southern Italy, 9 in central Italy and 20 in northern Italy. In our study, when GP trainees were asked if their Medical Training Course included a Global Health Course that included the topic of health protection and social and health care for migrant populations, 65 (33.8%) said answered “Yes,” 40 (20.8%) “No” and the largest percentage (45.3%) replied that they did not know. More than 60% of the GP trainees interviewed, therefore, have not attended or do not know whether a course with the aforementioned topic is included in the study plan and the data suggests that both at university and post-graduate training level the topic of global health and social and health care for migrant populations is not sufficiently taken into consideration, especially in Southern Italy (14). At the same time, when the GP Trainees were asked if they considered it useful within the Medical Training Course for General Practitioners to include a Global Health Course dedicated to the management of patients with a migratory background, most of the trainees (81.7%) answered “Yes.” Overall, the attendance of a Global Health Course that included the topic of healthcare for migrant populations is positively associated with the perception of being able to meet the health needs of the migrant population (OR = 3.34 95%CI 1.2–9.1; *p* = 0.018).

The need to structure courses dedicated to this topic from which GP Trainees can benefit is clear. In fact, among doctors who have dealt with patients with a migratory background in their clinical practice, the perception of having addressed the health needs of migrants is significantly associated with the attending of a Global Health Course focusing on the above-mentioned issues (*p* < 0.05). The questionnaire may have helped general practitioners understand how much of their education is overlooked, as they are aware of the usefulness of specific training on the above-mentioned issues.

Twenty-nine percent of the interviewed physicians believe that lectures are the most useful training experience, and at the same time consider live experience with a tutor the most useful learning method (52%). Similarly, a significant number of participants to a study conducted in Australia (15) focusing on Sexual and Reproductive Health (SRH) expressed a willingness to engage in further training and acknowledged the lack of training and knowledge in SRH to refugee and migrant women. A study assessing the experience, knowledge, and attitudes of GP trainees towards caring for refugees, asylum seekers, and undocumented migrants, conducted among 30 final year GP trainees in the UK revealed a significant lack of knowledge regarding migrants’ health needs and rights to care. The trainees also reported challenges in language barriers and a lack of experience and confidence in caring for this patient group (16). The identification of educational priorities in our study confirm the need for improved training on migrants’ health rights and effective communication strategies, as suggested by the UK study.

Suurmond et al. (17) identified specific cultural competences required by nurse practitioners (NPs) working with asylum seekers in the Netherlands. The key competences included knowledge of political and health situations in countries of origin, awareness of the effects of refugeehood on health, and the ability to use interpretation services. Similarly, our cross-sectional analytical pilot study highlighted a significant gap in the cultural competence of GPs attending the Medical Training Course.

In recent decades, in the WHO European Region, the health status of the population has improved a lot, but not equally. Ethnic minorities and some migrant groups and communities may suffer the most. The rapid increase in chronic diseases and mental disorders, the lack of social cohesion, environmental threats and financial uncertainties make improvement in health even more difficult and endanger the sustainability of health and welfare systems (18, 19). In Italy, regardless of citizenship and in accordance with art. 32 of the Constitution, all individuals are guaranteed access to the NHS (20). The foreigner who is regularly registered in our country is therefore equal to an Italian citizen in rights and duties if registered in the NHS. Conversely, foreigners in an irregular position, i.e., those without a regular residence permission or in any case without the required requirements, are guaranteed access to health services on a reduced basis, i.e., urgent and continuous outpatient and hospital care, support doctor regarding pregnancy, interventions to protect the health of the minor, vaccination and prophylactic interventions (21). These services are provided with the issuing of specific codes: Foreigner Temporarily Present (STP) for irregular non-EU foreigners; Non-Enrolled European (ENI) for EU citizens in poverty (22). During the COVID-19 pandemic, hundreds of thousands of people, including many migrants, found themselves excluded from protections, mitigation, and prevention programs (for example, swabs and vaccines), refreshments and, probably, also from recovery policies. Inequalities in the health sector for the migrant population must be considered “sentinel” events with respect to the effectiveness of integration policies and signal the urgency of improving the capacity to take charge of the health needs of an entire population (23).

Migration is recognized as a key determinant of health, affecting access to healthcare and interactions with healthcare workers. Furthermore, refugees and migrants face both formal barriers, complex legal rules and policy frameworks, and informal barriers, such as health literacy, low cultural competence of health workers, to achieving adequate healthcare (18). Within the questionnaire, one of the questions concerned the perception of healthcare professionals regarding the feeling of being able to meet the health needs of patients with a different cultural *background:* 60.8% of GP Trainees said they had the perception of meeting the health needs of patients with different cultural *backgrounds* “Sometimes” or “Rarely.” The unique health needs of the migrant population call for specific training of health professionals. In this respect, both having carried out clinical practice with patients with a migrant background (OR = 3.96 95%CI 1.7–9.2; *p* = 0.001) and having attended a Global Health Course (OR = 3.26 95%CI 1.1–9.2; *p* = 0.026) are positively associated with the feeling of being able to meet migrant patients’ health needs. The health needs of these populations have changed over time, increasingly approaching the needs of the host countries.

The dissemination of the document “*The Refugee and Migrant Health: Global Competency Standards for Health Workers (the Standards)*” offers a contribution to the debate on the education of GP Trainees regarding global health issues both nationally and internationally (4). It is important that this debate is continuously updated with new scientific contributions so that universities can formally recognize the importance of this approach for the education of future healthcare professionals. The cultural debate in this regard has also led to the strengthening of the art. 5 of the Italian Code of Medical Ethics “Health promotion, environment and global health.” The article currently states: “The doctor, in considering the living and working environment and the levels of education and social equity as fundamental determinants of individual and collective health, collaborates in the implementation of suitable educational, prevention and fight against health inequalities and promote the adoption of healthy lifestyles, providing information on the main risk factors. The doctor, based on available knowledge, works towards pertinent communication on exposure and vulnerability to environmental risk factors and promotes appropriate use of natural resources, for a balanced ecosystem that is also livable for future generations” (24).

Health professionals must be aware of the importance of the NHS and the legal frameworks that protect migrants in Italy, including their access to healthcare and what are the procedures that allow them to do so. As evidence of this, the inclusion of a Global Health Course dedicated to the management of patients with a migratory background within the Medical Training Course for General Practitioners is considered useful by the majority of respondents and significantly by female respondents (OR 3.0 95%CI 1.4–6.7; *p* = 0.005).

A study by Sørensen et al. (25) explored the essential topics and methods for a short, online course on diversity competence in healthcare. The participants rated the importance and urgency of various educational content areas and teaching methods. The consensus was that training should emphasize the health effects of migration, social determinants of health, and discrimination within the healthcare sector. Reflective practice was highly prioritized, with ‘reflection on own stereotypes and prejudices’ receiving significant consensus. These findings directly support the need identified by our study, targeting GPs, to enhance their cultural sensitivity and self-awareness. This alignment underscores the relevance of these findings in informing the design and implementation of targeted educational interventions to meet the specific needs identified in the Sicily pilot study.

As mentioned by Rajeshwari and Wright (26) in a study conducted in 2023, effective teaching not only prepares medical professionals to treat migrant patients but also fosters advocacy, sensitivity, empathy, and cultural competence, benefiting all underserved populations. As the first study of its kind, this study provided evidence to support integrating refugee healthcare into medical education, potentially guiding other medical schools, especially in countries hosting refugee populations.

Our research represents an initial exploration into the competencies of general practitioners in addressing the health needs of migrants and refugees. Consequently, the findings should be interpreted as preliminary conclusions. One significant limitation is that the questionnaire developed for this study, although tailored specifically to assess these competencies, has not undergone a comprehensive validation process. Therefore, the generalizability of the results is limited. There is a pressing need for further high-quality studies with larger, more diverse populations to confirm these findings and expand upon them. Comparative studies are also essential to understand how general practitioners’ competencies in this area compare across different regions and healthcare systems.

## 5 Conclusion

Refugees and migrants may have complex health needs due to the impact of migration and displacement on physical and mental health, as well as different health needs. They experience numerous difficulties in accessing healthcare: linguistic and cultural, discrimination and the limited availability of accessible, affordable, and appropriate healthcare services. These factors influence their interactions with healthcare professionals and the healthcare system, making it necessary to develop person-centred and culturally sensitive care. Health workers caring for refugees and migrants should be aware of how experiences of migration and displacement influence individuals’ health status and healthcare needs. Offering culturally sensitive care means that the healthcare provider applies their knowledge of the health of refugees and migrants, in such a way as to provide healthcare that meets individual health needs while adapting to the cultural context. Despite its limitations, our study provides important preliminary insights into the competencies of general practitioners in addressing the health needs of migrants and refugees in Sicily.

This is a critical and important topic, especially considering the new global challenges that characterize our times, such as the lack of social cohesion, climate change and financial uncertainties that aggravate health inequalities and which will contribute to an increase in migratory phenomenon. The issues that emerge from this study are diverse. On one hand, there is a need for future specialists to be prepared to assist a growing population. For this reason, training programs should integrate courses that address the specific health needs of migrants, as well as internships in contexts involving migrants. Additionally, activities such as specific continuing education courses or collaborations with public health experts can be planned. On the other hand, it is necessary that these actions are supported by specific clinical guidelines and effective integration health policies, also through funding and economic incentives. Another fundamental element concerns research in this specific area. Policy should also create inclusion programs that make access to healthcare truly equitable, such as public education programs or awareness campaigns.” Our study shows how we are still far from the standard competencies defined by the WHO that our doctors should have and how this is related to outdated training programs. Our research aims to highlight specific health needs and the necessity for adequate and updated training. From the questionnaires evaluated, it emerges that there is a need for clear information for the doctors of the future, who will increasingly deal with an expanding population with sometimes different needs. If the health of migrants and refugees is not preserved, health cannot be defined as global and, as doctors, we cannot, in good conscience, allow ourselves to define it as health.

## Data Availability

The raw data supporting the conclusions of this article will be made available by the authors, without undue reservation.
